# Supplementary figures and images for: Mental operations in rhythm: Motor-to-sensory transformation mediates imagined singing
Source: PLoS Biol. 2020 Oct 5;18(10):e3000504. doi: 10.1371/journal.pbio.3000504 (PMC7561264; doi:10.1371/journal.pbio.3000504)

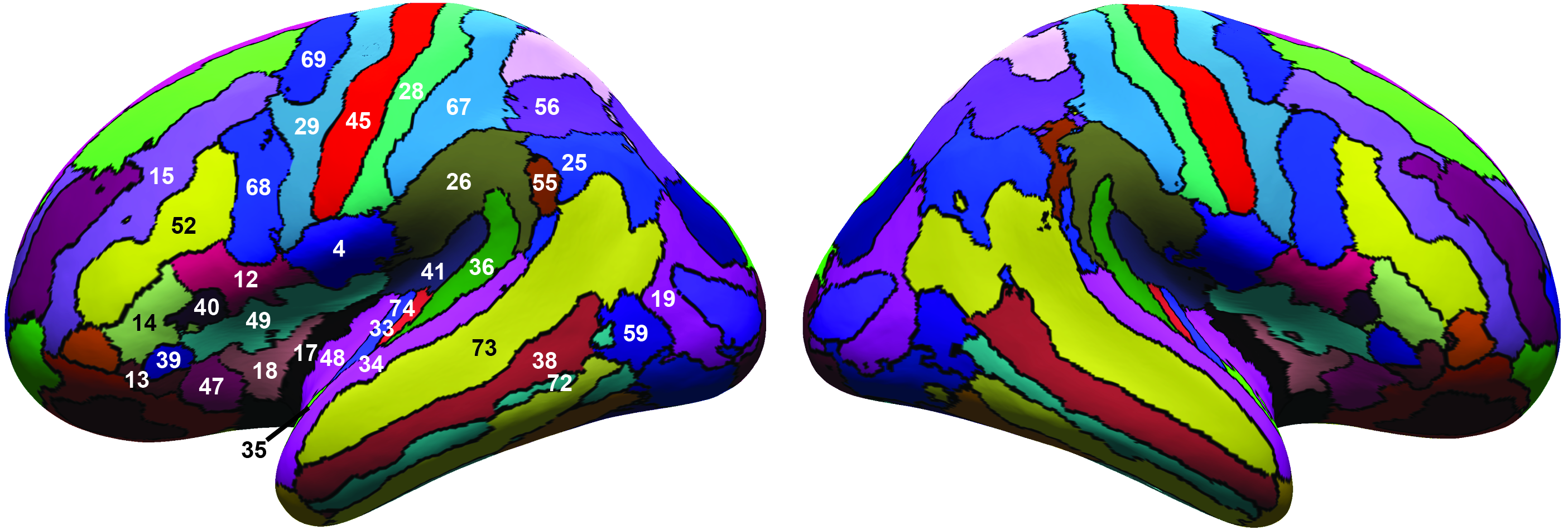

Supplement: S1 Fig — Parcellation superimposed on the inflated average cortical surface. Cortical areas related to this study were labeled with numbers on the left hemisphere. Refer to the S1 Table for the anatomical names for labels. The numeric labels were consistent with the ones used by Destrieux and colleagues [86]. (TIF) [file pbio.3000504.s001.tif]

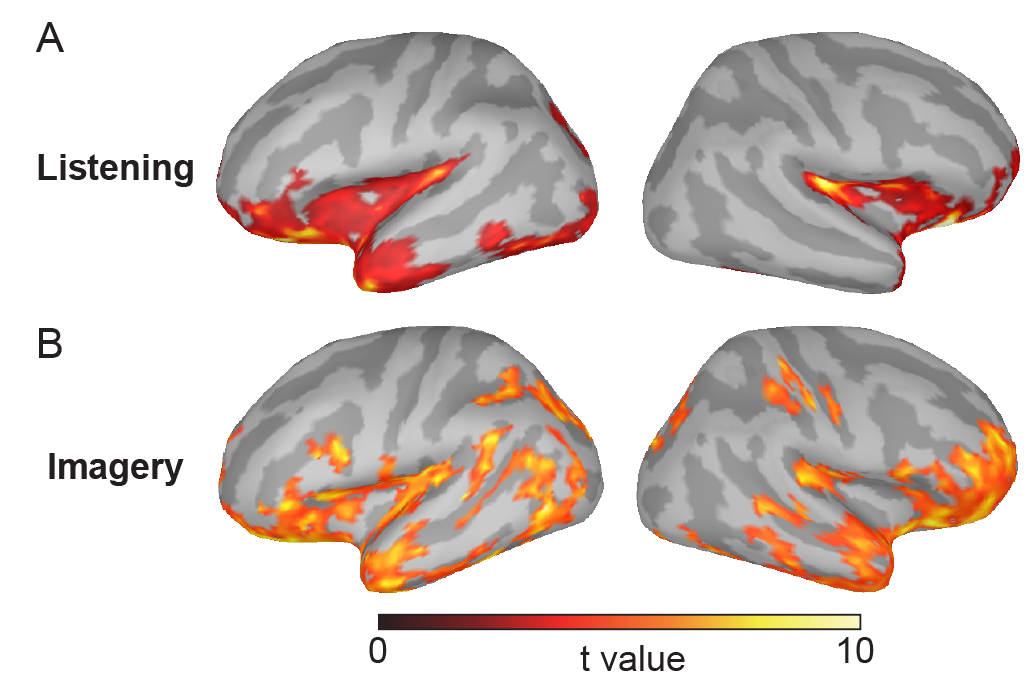

Supplement: S2 Fig — The results for (A) the listening condition and (B) the imagery condition were consistent with the results using 4-s-long epochs in Fig 2C and 2D. The underlying data for this figure can be found at https://osf.io/mc8wd/. (TIF) [file pbio.3000504.s002.tif]

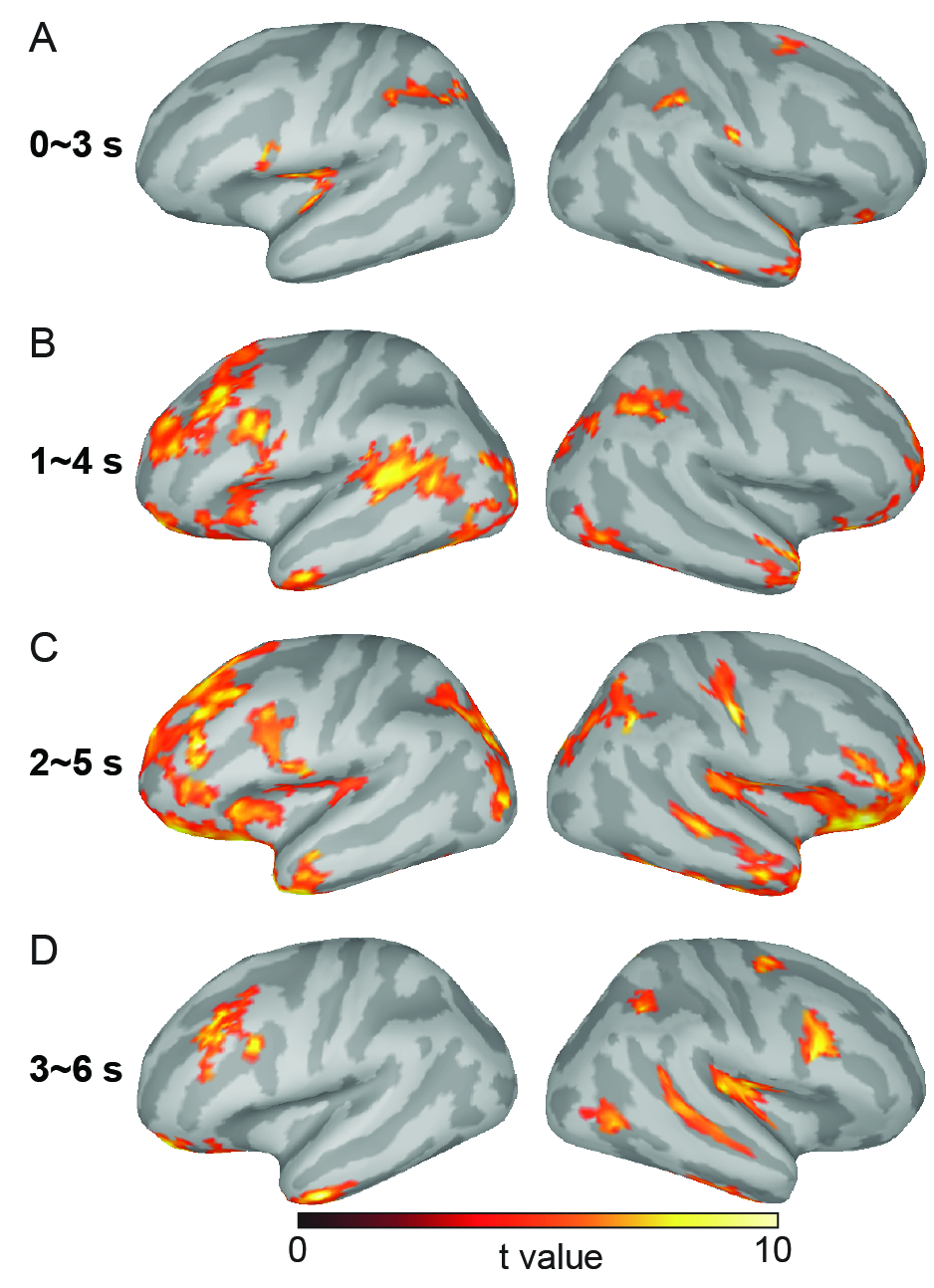

Supplement: S3 Fig — The results were more reliably toward the end of trials and were consistent with the results in Fig 2D. The underlying data for this figure can be found at https://osf.io/mc8wd/. (TIF) [file pbio.3000504.s003.tif]
