# Supplementary material for: Mental operations in rhythm: Motor-to-sensory transformation mediates imagined singing
Source: PLoS Biol. 2020 Oct 5;18(10):e3000504. doi: 10.1371/journal.pbio.3000504 (PMC7561264; doi:10.1371/journal.pbio.3000504)
Supplement: S1 Table — (DOCX) [file pbio.3000504.s004.docx]

| Index | Short name | Long name |
| --- | --- | --- |
| 4 | G_and_S_subcentral | Subcentral gyrus (central operculum) and sulci |
| 12 | G_front_inf-Opercular | Opercular part of the inferior frontal gyrus |
| 13 | G_front_inf-Orbital | Orbital part of the inferior frontal gyrus |
| 14 | G_front_inf-Triangul | Triangular part of the inferior frontal gyrus |
| 15 | G_front_middle | Middle frontal gyrus (F2) |
| 17 | G_Ins_lg_and_S_cent_ins | Long insular gyrus and central sulcus of the insula |
| 18 | G_insular_short | Short insular gyri |
| 19 | G_occipital_middle | Middle occipital gyrus (O2, lateral occipital gyrus) |
| 25 | G_pariet_inf-Angular | Angular gyrus |
| 26 | G_pariet_inf-Supramar | Supramarginal gyrus |
| 28 | G_postcentral | Postcentral gyrus |
| 29 | G_precentral | Precentral gyrus |
| 33 | G_temp_sup-G_T_transv | Anterior transverse temporal gyrus (of Heschl) |
| 34 | G_temp_sup-Lateral | Lateral aspect of the superior temporal gyrus |
| 35 | G_temp_sup-Plan_polar | Planum polare of the superior temporal gyrus |
| 36 | G_temp_sup-Plan_tempo | Planum temporale or temporal plane of the superior temporal gyrus |
| 38 | G_temporal_middle | Middle temporal gyrus (T2) |
| 39 | Lat_Fis-ant-Horizont | Horizontal ramus of the anterior segment of the lateral sulcus (or fissure) |
| 40 | Lat_Fis-ant-Vertical | Vertical ramus of the anterior segment of the lateral sulcus (or fissure) |
| 41 | Lat_Fis-post | Posterior ramus (or segment) of the lateral sulcus (or fissure) |
| 45 | S_central | Central sulcus (Rolando's fissure) |
| 47 | S_circular_insula_ant | Anterior segment of the circular sulcus of the insula |
| 48 | S_circular_insula_inf | Inferior segment of the circular sulcus of the insula |
| 49 | S_circular_insula_sup | Superior segment of the circular sulcus of the insula |
| 52 | S_front_inf | Inferior frontal sulcus |
| 55 | S_interm_prim-Jensen | Sulcus intermedius primus (of Jensen) |
| 56 | S_intrapariet_and_P_trans | Intraparietal sulcus (interparietal sulcus) and transverse parietal sulci |
| 59 | S_occipital_ant | Anterior occipital sulcus and preoccipital notch (temporo-occipital incisure) |
| 67 | S_postcentral | Postcentral sulcus |
| 68 | S_precentral-inf-part | Inferior part of the precentral sulcus |
| 69 | S_precentral-sup-part | Superior part of the precentral sulcus |
| 72 | S_temporal_inf | Inferior temporal sulcus |
| 73 | S_temporal_sup | Superior temporal sulcus (parallel sulcus) |
| 74 | S_temporal_transverse | Transverse temporal sulcus |
