# Supplementary material for: Mental operations in rhythm: Motor-to-sensory transformation mediates imagined singing
Source: PLoS Biol. 2020 Oct 5;18(10):e3000504. doi: 10.1371/journal.pbio.3000504 (PMC7561264; doi:10.1371/journal.pbio.3000504)
Supplement: Attachment — Submitted filename: responses_imagined_singing_MEG_20200622-final.docx [file pbio.3000504.s005.docx]

Dear Dr. Gasque,

We thank you and the reviewers for the constructive comments. We are glad to hear that all reviewers acknowledge the technical solidness and potential interests of our study. According to the detailed suggestions, we have substantially revised our manuscript. The major updates include

1) Provided an explicit rationale of the predetermined sample size. We have demonstrated that our selection of sample size followed rigorous scientific procedures as suggested by reviewer 3. Moreover, we have calculated the post-hoc effect sizes that are larger than the required theoretical effect size. Together with significant results in different analyses, these evidence support that the study has enough statistical power;
2) Reframed and broadened the research question to mental operations of rhythmicity and tonality by including both inner speech and covert singing. The updated questions as suggested by reviewer 3 fully exploit the results and new analyses, and motivate the investigation of this study in a broader cognitive domain;
3) Clarified the novelty and significance of this study by emphasizing the investigation of neural dynamics;
4) Performed the phase coherence analysis in five more frequency bands as suggested by reviewer 1. These new analyses provide crucial evidence suggesting the neural dynamics in the motor-to-sensory transformation and hence boost the novelty and significance of this study. Moreover, the new results support the reframed research questions that concern the mental operations related to speech and singing.
5) Conducted direct comparisons between listening and imagery;
6) Carried out a conjunction analysis and revealed the common regions that mediated listening and imagery;
7) Tested the reliability of our results using longer epochs. The new results are consistent with the original results.
8) Reran the phase coherence analysis with four songs. The results are consistent with our hypothesis.
9) Investigated the evolution of phase coherence across time. The results are consistent with the original results.
10) Provided clarifications throughout the manuscript.

Our heavily revised manuscript includes two additional main figures, three supplementary figures, and one supplementary table. We believe that our manuscript has been significantly improved. Please find our point-to-point responses in **bold** below each comment.

Sincerely,

Xing Tian

(on behalf of Yanzhu Li and Huan Luo)

p.s. We are extremely grateful for having such dedicated editors and reviewers. We really appreciate the constructive comments and everyone’s hard work, especially during these challenging times. We hope that everyone stays healthy and sound.

Reviewer #1: The study by Li and colleagues investigated whether imagined singing was associated with activation of the motor-to-sensory-transformation network, as quantified by the M100 response and changes in phase coherence in the delta frequency band (which corresponded to the approximate rhythm of the song frequency). They reported that imagined singing produced changes in measures that were comparable (in pattern and magnitude) to those elicited by passive listening to the songs. This study follows on from the team’s influential work on the role of the motor-to-sensory transformation network in inner speech. The design was creative and innovative; I particularly liked the idea of frequency tagging the rhythm of inner speech (discussed further below).

I have some questions and comments regarding the methodology and results that the authors could consider:

**We thank the reviewer for acknowledging the significance and novelty of this study. According to the constructive suggestions, we have provided thorough clarifications about our paradigm, methods, and results. Moreover, we have conducted additional analyses as suggested. Especially, the suggested investigation in more frequency bands yields interesting results that further reveal the dynamics in the motor-to-sensory transformation. (We really appreciate these suggestions.) Please see our detailed responses below.**

-	If I could just clarify my understanding of the experimental design: participants were first played the full song (~ 5 – 8 seconds – these constituted the ‘listening trials’). There was then a gap of 1.5 seconds. Then the fixation cross changed color and participants were required to imagine themselves singing the song (these constituted the ‘imagery trials’). Is this correct?

**Yes. The reviewer understood correctly. We have added more clarifications in the Methods section.**

-	The similarity in the form and magnitude of the M100 elicited by the song onset in the listening condition and the imagery onset in the imagery condition is impressive, and the authors’ claim that the auditory cortices were activated in the imagery conditions seems plausible (to me). However, is it possible that this component was (partly) elicited by the change in color of the fixation cross? It would presumably be easy to discount this possibility by measuring the MEG response to a colour change presented alone (i.e., in the absence of auditory stimulation or song imagery).

**We agree with the reviewer that the co-occurrence of color change in fixation and the onset of imagery could elicit a combination of visual and auditory neural responses. However, we believe the observed M100 response in the imagery is less likely caused by the color change of fixation. The M100 responses in hearing and imagery were very similar (Original Fig.1 g&h, the new Fig. 2 A&B). Both of them demonstrated a canonical auditory response topography in which a dipole pattern was over temporal lobe region in each hemisphere [e.g. Roberts, T. P., & Poeppel, D. (1996). Latency of auditory evoked M100 as a function of tone frequency. *NeuroReport*, *7*(6), 1138-1140.]. This response topography contrasts with early visual responses in MEG that show more posterior dipole patterns [e.g. Liu, J., Harris, A., & Kanwisher, N. (2002). Stages of processing in face perception: an MEG study. Nature neuroscience, 5(9), 910-916; Dikker, S., Rabagliati, H., & Pylkkänen, L. (2009). Sensitivity to syntax in the visual cortex. Cognition, 110(3), 293-321.].**

**The reviewer’s suggestion is a very good way to tease apart the different perceptual responses between different modalities. Unfortunately, we did not have the capacity to run recently. During designing our experiment, we kept the possible visual impact in mind and explicitly used the color change as a visual cue, instead of the onset of any visual cues. Generally, the onset of visual stimuli can induce a large early perceptual response, compared with color change. In this way, we can minimize the influence of visual responses. The precaution in experimental design and the auditory-like M100 response patterns in imagery suggests that this early ERP component is more likely caused by the onset of imagined signing. We have added clarifications in the Methods and Results sections and copied below.**

**[‘Both M100 responses demonstrated a canonical auditory response topography in which a dipole pattern over the temporal lobe region in each hemisphere was observed (e.g., Roberts & Poeppel, 1996). This response topography contrasted with earlier visual responses in MEG that showed more posterior dipole patterns (e.g., Dikker, Rabagliati, & Pylkkänen, 2009; J. Liu, Harris, & Kanwisher, 2002). Therefore, the M100 responses in both the listening and imagery conditions were less likely to be induced by the fixation color changes.’]**

-	Regarding the temporal domain (waveform) analysis: I don’t understand why only the first sound (or sound image) of the song elicited an M100 – can the authors clarify? If this signal truly reflects the activation of the auditory cortex by the imagined song, wouldn’t each individual letter be expected to elicit an M100? If might be useful to illustrate the onset of each individual letter in the listening condition (e.g., by means of a dotted line), to see if each letter is followed by a M100 (albeit smaller than the M100 elicited by the first letter). Relatedly, in the Results the authors state: “Moreover, no repetitive patterns were observed in the time course of listening or imagery (Fig. 1g&h), suggesting that the tracking of the acoustic stream or the rate of imagery was not by the response magnitude” – as above, I don’t understand why the authors were not expecting a repetitive pattern if each specific ‘sound image’ was associated with an M100, and particularly given that they claim to have identified delta oscillations to the inner song. Can the authors clarify why they would expect the first sound image in the sound to elicit an M100, but not subsequent sound images? An alternative explanation is that that the signal actually reflects an ‘omission M100’ response (akin to the oN1 response in the ERP literature) caused by a violation in the expectation of hearing a sound – this could perhaps explain why the signal is only present to the first sound in the song. I would be interested to hear the authors’ thoughts on this.

**The reason for the absence of M100 for each sound is probably due to the repetition suppression [Grill-Spector, K., Henson, R., & Martin, A. (2006). Repetition and the brain: neural models of stimulus-specific effects. Trends in cognitive sciences, 10(1), 14-23.]. Usually, the onset of the first stimuli induces a large response. Whereas as the number of repetitions increases, the response magnitude goes down and plateaus. It is evident in the dynamics of responses in listening condition (original Fig. 1g, the new Fig. 2A) -- the M100 to the first sound had a large peak, but the onset responses to the following sounds are not apparent. The same situation applies to the imagery condition. Moreover, the magnitude of imagery responses is smaller than the perceptual responses [e.g. Tian & Poeppel, 2010 Frontiers in Psychology]. Furthermore, the active nature of imagery could reduce the event-related response compared to hearing. The first onset of imagined sound could align with the onset cue. However, the temporal variance arguably becomes larger as the imagined process progresses. Therefore, the event-related responses could become even smaller after averaging across trials. That is why it is hard, if not impossible to provide precise time-locked responses to every onset of imagined sound. We provided an intermediate solution by showing the raw data with RMS waveform for the dynamics and topographies at every 333ms for the close looks of amplitude distribution that presumably associated with the frequency of interest. Furthermore, we overcome this temporal variance problem in imagery with a novel method that compares between trials with different degrees of temporal variance to reveal the dynamics and possible neural implements for mental imagery.**

**Previous studies on neural tracking to speech have demonstrated a similar phenomenon -- the consistency across trials was demonstrated in temporal features rather than response amplitude. For example, the neural entrainment to continuous speech signals is manifested in the neural phase, but not in amplitude [Luo, H., & Poeppel, D. (2007). Phase patterns of neuronal responses reliably discriminate speech in human auditory cortex. Neuron, 54(6), 1001-1010.]. This is probably because the repetition suppression makes the measure of amplitude less reliable, while the timing information (neural phase) is independent of amplitude, and is less influenced by repetitions and more sensitive to reflect the dynamics. Therefore, we took advantage of sensitivity in the neural phase to reveal the dynamics and possible neural implementations of speech imagery.**

**The reviewer correctly pointed out that the omission of sounds can elicit auditory-like responses. However, because of the paradigm and the nature of imagery tasks, we believe the observed M100 to the onset of imagery is less likely to be caused by the violation of the expectation of sound. First, the cue of imagery always occurs 1.5s after the offset of songs. Participants were explicitly told to start imagining when they saw the cue. This contrasts with the omission paradigm in which the omission is unexpected occurred immediately after a sequence of regular sounds. Moreover, the omission is in passive listening conditions, whereas in the current study, the imagery is an active construction process. Therefore, the observed imagery-induced M100 is likely generated from a different mechanism from the one mediating omission.**

**We have added all these points in the Results and Discussion sections and copied below.**

**[‘No repetitive topographic patterns were observed in the time course of listening or imagery (Fig. 1A&B), suggesting that tracking of the acoustic stream or the rate of imagery was not by the response magnitude. These results are consistent with previous studies that showed the absence of power coherence in speech tracking (Luo & Poeppel, 2007). The lack of effects in response magnitude was probably due to repetition suppression (Grill-Spector, Henson, & Martin, 2006). Repetitions of similar processes decreased the response magnitude. Moreover, the temporal variance in imagery could further reduce the magnitude of evoked responses. Therefore, we further investigated the timing information and neural tracking of the induced responses in the spectral domain.’]**

-	Regarding the time-frequency (spectral) analysis: I think it would be helpful if the authors could add plots showing the change in phase coherence across time, and across a range of frequencies (i.e., demonstrate that the inner singing elicited oscillations). Without this information, how can the authors be sure that the ‘entrainment’ they report is specific to the delta band (i.e., the approximate frequency of the rhythm)? While their hypothesis is plausible, it seems to me first necessary to show that the entrainment is specific to delta, and does not also occur in other frequency bands (theta, alpha, beta, gamma, etc.).

**This is a very good idea. According to this excellent suggestion, we have run additional analyses on the theta (4-7 Hz), alpha (8-12 Hz), low-beta (13-16 Hz), mid-beta (17-20 Hz), high-beta (21-28 Hz), and low gramma (30-48 Hz) bands. The new results reveal very interesting patterns and frequency band-specific distribution, which are consistent with auditory and motor control literature, suggesting that specific frequencies operate particular functions in the motor-to-sensory transformation. We have added these new results as Fig. 3, and added texts in the Methods, Results and Discussion sections, and copied below.**

**In Results, [‘To further explore the functional specificity of dynamic processing in the motor-to-sensory pathway, we performed the phase-coherence analysis in a broad range of frequency bands [Fig. 3]. We found that in the 4-7 Hz (the theta band), only the primary auditory cortex and anterior part of STG and STS in the left hemisphere showed significant phase coherence. This focused theta band activation in the auditory cortices, contrasted with the collaborative frontal-parietal-temporal network activation of the entire motor-to-sensory pathway in the delta band (Fig. 1), was consistent with the specific role of the theta band in auditory and speech processing (e.g., Teng, Tian, Doelling, & Poeppel, 2018; Teng et al., 2017).**

**A broad sensorimotor network in the right hemisphere was observed in 8-12 Hz (the alpha band, or the ‘mu’ rhythm) and 16-20 Hz (the mid-beta band). These frequency bands also showed higher phase coherence in the inferior parts of subcentral gyrus and opercular part of the inferior frontal gyrus in the left hemisphere; The 16-20 Hz also extended to the premotor cortex. The specific involvement of sensory and motor systems in the mu and beta bands were consistent with the alpha-beta synchronization in motor control and motor imagery (e.g., McFarland, Miner, Vaughan, & Wolpaw, 2000; Pfurtscheller, Brunner, Schlögl, & Da Silva, 2006), suggesting the specific dynamics for the motor simulation and somatosensory estimation in the motor-to-sensory transformation (Tian & Poeppel, 2010, 2012; Tian et al., 2016). The right hemisphere dominance was consistent with imagery in a singing context (e.g., Özdemir, Norton, & Schlaug, 2006; Riecker, Ackermann, Wildgruber, Dogil, & Grodd, 2000; Zarate, 2013).**

**In the low-gamma band, greater synchronization was observed in the center-group than that in the dispersed-group over the entire motor and sensory systems that showed tracking of imagery in the delta band (Fig. 2D). These results are consistent with the view of local computations for the gamma band (Buzsáki & Wang, 2012; Kopell, Ermentrout, Whittington, & Traub, 2000). The distinctive neural involvement in the tracking rate of the delta, as well as in the theta, mu, beta, and gamma bands, collaboratively indicates the extent of the motor-to-sensory transformation network and the specific functional dynamics of each frequency component in this network.’]**

**In Discussion, [‘The observations of neural structures that operate at separate frequency bands are consistent with our hypothesis regarding the algorithms and neural implementations in the motor-to-sensory transformation. First, using songs with a rhythm in the delta range, a complete frontal-parietal-temporal network was revealed in the corresponding low-frequency band during the imagined singing tasks [Fig. 2D]. These results provide functional evidence suggesting the extent of the motor-to-sensory transformation pathway, consistent with findings in neuroimaging studies (Hurlburt, Alderson-Day, Kühn, & Fernyhough, 2016; McGuire et al., 1995; McGuire et al., 1996). More importantly, the neural phase tracking in the delta band suggested that the fluctuation of the excitability in the network temporally aligned with the unfolding of mental operations.**

**Second, phase coherence at the theta band was localized at temporal auditory regions [Fig. 3A]. These results are consistent with the view that theta oscillations actively mediate the auditory and speech processes (Teng et al., 2018; Teng et al., 2017). In the context of motor-to-sensory transformation, the precise localization of the theta band suggested that theta oscillations could mediate specific computations for constructing speech-like representations.**

**Third, the mu and beta bands have indicated neural rhythmic signatures for both motor control and imagery (e.g., McFarland et al., 2000; Pfurtscheller et al., 2006). In this study, we observed a broad sensorimotor network operating in the mu and beta rhythms during the imagined singing tasks [Fig. 3B&D]. These results further suggested that computations could be executed at these rhythms to internally simulate action during imagery (Li et al., 2020; Ma & Tian, 2019; Tian & Poeppel, 2010, 2012; Tian et al., 2016). Such motor simulations presumably generate an efference copy that is used to emulate the somatosensory consequences of actions for estimating the status of motor effectors (Tian & Poeppel, 2010, 2012; Tian et al., 2016).**

**The hemispheric lateralization highlighted the commonalities and distinctions between inner speech and covert singing. The bilateral observations in the delta band supported neural entrainment to the common rhythmicity in both types of mental operations. The lateralized theta band in the left auditory cortices was consistent with speech-weighted processing (Giraud & Poeppel, 2012). Whereas, the right-hemispheric dominance in the mu and beta bands over the sensorimotor cortices suggested the potential construction of melodic and tonal information in the singing context (e.g., Özdemir et al., 2006; Riecker et al., 2000; Zarate, 2013). The switching from right to left hemisphere is consistent with the observations that the contents of auditory information determine the hemispheric lateralization –the right-lateralized processing of the humming tones is switched to the left lateralization when the tones are superimposed on syllabic contents (Mei, Flinker, Zhu, Cai, & Tian, 2020).**

**Fourth, the gamma band was observed over the entire motor and sensory systems. This result indicated the implementation of local computations in modality-specific regions (Buzsáki & Wang, 2012; Kopell et al., 2000). Together, the motor-to-sensory transformation network possesses distinct dynamics for mediating mental operations. The frontal-parietal-temporal network entrains the rhythm of metal operations. This low-frequency entrainment is suggested to facilitate communication and coordination between cortical areas for specific tasks via traveling waves (Arnal, Doelling, & Poeppel, 2015; Muller, Chavane, Reynolds, & Sejnowski, 2018; Nácher, Ledberg, Deco, & Romo, 2013). Moreover, via cross-frequency coupling (Buzsáki & Wang, 2012), specific frequencies (theta for auditory, mu and beta for sensorimotor) may modulate the gamma band to achieve the computations of motor simulation as well as somatosensory and auditory estimation in the motor-to-sensory transformation in the imagery tasks (Giraud & Poeppel, 2012; Kopell et al., 2000). Future studies are necessary to investigate these hypotheses regarding the connectivity across frequencies and cortical regions so that the functional contributions of the proposed model can be thoroughly examined.’]**

**Moreover, according to the constructive suggestion, we tested how the phase coherence changed across time. Four 3s time bins (0-3s, 1-4s, 2-5s, and 3-6s) were created in longer time epochs (0-6s). The phase coherence was estimated in each of the four time-bins. The results are consistent with the major original findings of the frontal-parietal-temporal network, although these results have less power because of a shorter period in each time bin. Moreover, these results suggest that the phase coherence in imagery requires time to establish, as the results become more reliable in later time bins. We have included these new results in supplementary information S3 Fig. and copied below.**

**[‘Moreover, the analysis of four 3s consecutive time bins revealed that the entrainment to imagery required time to be established, as the phase coherence differences became more stable in the later time bins [S3 Fig.]’]**

-	I thought the ‘frequency-tagging’ aspect of the design was interesting and creative. However, given that the rhythm was approximately equivalent for all of the songs (and the data were averaged across songs in any case) I feel like this aspect of the design could be developed further. For example, it would be interesting to compare the oscillations elicited by ‘delta rhythm’ songs vs. ‘theta rhythm’ songs, to see if the oscillations they elicit are specific to their rhythm: a sort of ‘auditory steady-state response’ for imagined sounds.

**We thank the reviewer for acknowledging the novelty of our study. It is really a good idea to use different speeds of songs to demonstrate the analogy of ‘auditory steady-state response’ in imagery. This would provide even stronger evidence for the neural entrainment to speech imagery. The reason we chose the delta rhythm in this study is that the fundamental differences between perception and production. In perception, auditory processing can track a range of frequencies in the stimuli from low (e.g. the low delta band) to high (e.g. the gamma band). However, imagery is an active constructing process. According to our hypothesis, the speed of speech imagery is constrained by the motor system -- the intrinsic network for production operates at a speed that is similar to the speed of physical movement of articulation apparatus. Arguably, the faster the imagery, the harder the task. That is, the higher the speed of production, the more temporal variance would be. As demonstrated in songs, especially for the familiar nursery rhymes, the most natural speed is about in the delta band. Therefore, we chose the delta range to increase power in the first attempt of a novel approach. We thank the reviewer’s excellent suggestion and will explore more possibilities of novel experimental designs in future research.**

-	I’m not sure I fully understand the rationale for comparing the ‘center-group’ (which would more accurately be labelled as ‘center-trials’) and the ‘dispersed-group’ in the imagined song analysis – is the idea that the motor-based predictions are better in the ‘center-trials’, as the RTs better matched the duration of the actual song? If so, I’m not sure I agree – given that participants overestimated the duration of the song in all imagery conditions, doesn’t this imply that the trials with the lowest RTs actually reflect the most accurate predictions? But in any case, why would the ‘motor-based-predictions’ be expected to be stronger in the center trials? What is the benefit of this approach against, say, simply comparing the ITC when (a) participants listened to songs, (b) participants produced inner songs, and (c) participants sat passively. Presumably the (b) vs. (c) comparison would be expected to be even stronger in such an analysis?

**The ‘center-group’ and ‘dispersed-group’ were not defined by matching the RTs to the durations of actual songs. They were defined as compared to the mean of RTs in the imagery task. The RT differences among trials in the ‘center-group’ were much smaller than those among the trials in the ‘dispersed-group’ (Fig. 1d). The similar durations indicated that trials in the ‘center-group’ were more likely imagined in a similar temporal manner. According to our neural entrainment hypothesis, the neural phase patterns would be more consistent in the ‘center-group’ compared with the ‘dispersed-group’, and hence higher ITC value. The trials with the lowest RTs would be closer to the duration of the actual song. But when calculating ITC among imagery trials, the lowest RT trials would have the largest temporal variance, and hence the lowest ITC.**

**We implemented comparisons between ‘center-group vs. dispersed group’ to overcome difficulties in investigating speech imagery that was induced by the nature of imagery tasks. The uniqueness of imagery is its active nature. Unlike passive hearing in which every trial has a fixed duration, imagery performance varies from trial to trial. Based on established ITC calculation using all trials in imagery would reduce the power that is already very small in imagery. The split-group is a development of the ITC methods by adapting to the unique requirement of imagery. Furthermore, the intrinsic baseline (rest) could lack control and involve additional confounding factors. Whereas, trials in the dispersed-group had identical procedures and tasks. The only difference from trials in the center-group was the responses that reflected the measure of interest (the dynamic processes during imagery). Therefore, the dispersed-group is a better control. Using the temporal differences while maintaining everything else consistent can precisely reveal the dynamics of interest in the mental imagery tasks. We have added these clarifications in the Methods section and copied below.**

**[‘Because the separation of trials was defined by the differences between individual trial RT and the mean RT in the imagery condition, the RT differences among trials in the center-group were much smaller than those among the trials in the dispersed-group [Fig. 1D]. Similar durations indicated that trials in the center-group were more likely imagined in a similar temporal manner. These temporal differences between groups of trials were used in the phase coherence analysis of MEG to investigate our hypothesis of neural entrainment to imagery.’]**

-	With regards to the source-localization: why did the critical p-values differ between the listening and imagery conditions – did the number of statistical comparisons differ between these conditions? As an aside, while the required number of trials per condition is a matter of debate, 12 trials per condition seems pretty minimal for a paradigm with no external stimulation where a large effect size would presumably not be expected – can the authors comment?

**In the previous version, the p-values differed between the listening and imagery conditions because different statistical procedures were used. In the listening condition, first, a random sample grouping of between-groups was conducted 100 times to increase the reliability of generated baselines. The 95th percentile of ITC values was selected as a baseline value (already a threshold of 0.05) to contrast with the empirical value in the within-groups. Therefore, the p-value 0.05 was selected in the following cluster analysis for the listening condition. Whereas, in the imagery condition, the center-group was directly compared with the dispersed-group in the cluster analysis, so that p-value of 0.001 was used. These above-mentioned p-values are all pre-cluster level threshold. (We did not make it clear in the previous version, and we have clarified in the updated version.) The cluster-level threshold for both conditions is FDR corrected p < 0.05. (The p_corr(FDR) < 0.01 in the main text of the previous version was a typo and we have corrected it.)**

**We agree with the reviewer that the different p-values were confusing. Therefore, in the updated version, we used a similar statistical procedure and the same pre-cluster p-values of 0.001 and cluster level p-values of 0.05 in the cluster analysis for both the listening and imagery conditions. Specifically, we selected the median ITC value (50th percentile) in the 100 random sample grouping of between groups as a baseline value in the listening condition, instead of the 95th percentile. The same original procedure was used in the imagery condition. Therefore, the same pre-cluster p-values of 0.001 and cluster level p-values of 0.05 were used in both the listening and imagery conditions. The new results were consistent with the original results.**

**Regarding the trial numbers, each trial in the imagery condition had at least 4 second long data for analysis. FFT was applied with a window of 500ms with a moving step of 200ms, yielding 19 data points in each trial. Each group included 12 trials with 19 data points in each trial. This should be enough for estimating the phase coherence, as the positive results in multiple analyses suggested.**

**All these points have been added to the Method section and copied below.**

**[‘Fast-Fourier-transform (FFT) was applied to each trial in a group with a 500ms time window in steps of 200ms, yielding 19 time points in each 4s-long trial epoch.’]**

**[‘The empirical statistics were first obtained by a two-tailed paired t-test with two or more adjacent significant vertices with a pre-cluster threshold of alpha = 0.001. Next, a null distribution was formed by randomly shuffling the group labels 1000 times. Cluster-level, FDR corrected results were obtained by comparing the empirical statistics with the null distribution (cluster threshold of alpha=0.05 for both listening and hearing conditions).’]**

Reviewer #2: In « Verbal thinking in rhythm: motor-to-sensory transformation network mediates imagined singing », Li and colleagues aimed to define the brain networks associated to imagined singing with an elegant design and methodological approach (frequency tagging on MEG data).

The study is interesting and rigorously performed using state-of-the art analyses tools. However I am wondering about the novelty and significance of the findings, as the implication of fronto-temporo-parietal networks in speech and music mental imagery is already very well established (as pointed out by the authors in the introduction and discussion sections). Moreover the potential novelty of this study, as stated by the authors (naturalistic sounds, frequency tagging approach, and MEG source localization), is mainly methodological and is, in my opinion, not so novel because these approaches have been used by plenty of research groups in the domain.

In its current form, I thus think if this report might be more suitable for a more specialized journal.

**We thank the reviewer for acknowledging the reliability, general interests, and advanced techniques of our study. We believe that the novelty of our study is the investigation of dynamics. The investigation of motor-to-sensory transformation is highly significant as indicated in a broad range of research including motor control, visual perception, speech production, as well as higher-level cognitive functions such as mental imagery. Most previous studies used neuroimaging techniques and focused on revealing the anatomical locations of this function, such as the implication of frontal-parietal-temporal networks in speech and music mental imagery [e.g. Zatorre & Halpern, 2005 *Neuron*; Kraemer et al., 2005 *Nature*; Tian, Zarate, & Poeppel, 2016 *Cortex*; Tian et al., 2018 *Nature Human Behaviour*]. However, neural dynamics in this network are still unclear. Dynamics provide important information about neural computations in addition to anatomical locations [Buzsáki & Draguhn, 2004 *Science*; Carandini, 2012 *Nature Neuroscience*]. For example, studies that investigate neural dynamics in well-known localized speech and language areas are still providing break-throughs and are published in prestigious journals [e.g. Anumanchipalli, Chartier, & Chang, 2019 *Nature*; Makin, Moses, & Chang, 2020 *Nature Neuroscience*]. The current study, to the best of our knowledge, is the first attempt to collectively reveal the dynamics in the entire proposed network that is hypothesized to mediate mental imagery of speech and singing. Our study potentially, from a dynamic perspective, provides a novel way to imply the functions and computations in the motor-to-sensory transformation.**

**Probing dynamics is difficult, especially the ones that mediate pure mental operations without external stimulations. The active nature of mental tasks inevitably causes temporal variations that undermine the foundations of methods for probing dynamics. For example, the phase coherence analysis in the frequency tagging approach is well established for speech perception research. But the same analysis cannot directly apply to investigate the neural entrainment of speech imagery -- the within-group would have too much temporal variance across trials and hence the phase coherence would be too small to detect. In this study, we overcame the obstacles by taking advantage of the temporal variance -- separating trials based on behavioral timing. This methodological advance, in the combination of naturalistic sounds and source localization, makes it possible to investigate the dynamics in neural networks that mediate mental operations.**

**Our results suggest possible functions in the networks. The original results of neural phase tracking in the delta band suggest that the fluctuation of the excitability in the network temporally aligned with the unfolding of mental operations. Moreover, we have run additional analyses in more frequency bands as suggested by reviewer 1. The new results (the new Fig. 3 and copied below) further demonstrate a double dissociation of operating frequencies and anatomical locations. The theta band was observed over left auditory cortices, consistent with the speech processing. Whereas the mu and beta bands were observed in the sensorimotor systems in the right hemisphere, consistent with the melodic processing in singing. This double dissociation hints the possible different computations in inner speech and covert singing in the proposal motor-to-sensory transformation pathway. Such rich dynamics cannot be obtained in any neuroimaging studies. We have copied the new results and discussions below.**

**Together, our endeavors in exploring neural dynamics of mental operations make positive theoretical, methodological, and empirical contributions. We believe that our study fits with the ‘novel and interest’ aim of the short reports in PLOS Biology: ‘simply apply known elegant techniques to elucidate a brief answer to an interesting scientific question’ (**[**https://journals.plos.org/plosbiology/article?id=10.1371/journal.pbio.3000248**](https://journals.plos.org/plosbiology/article?id=10.1371/journal.pbio.3000248)**). In the updated version, we have more explicitly stated the significance and novelty of our study throughout the manuscript.**

**Copies of the new results and discussions below.**

**In Results, [‘To further explore the functional specificity of dynamic processing in the motor-to-sensory pathway, we performed the phase-coherence analysis in a broad range of frequency bands [Fig. 3]. We found that in the 4-7 Hz (the theta band), only the primary auditory cortex and anterior part of STG and STS in the left hemisphere showed significant phase coherence. This focused theta band activation in the auditory cortices, contrasted with the collaborative frontal-parietal-temporal network activation of the entire motor-to-sensory pathway in the delta band (Fig. 1), was consistent with the specific role of the theta band in auditory and speech processing (e.g., Teng, Tian, Doelling, & Poeppel, 2018; Teng et al., 2017).**

**A broad sensorimotor network in the right hemisphere was observed in 8-12 Hz (the alpha band, or the ‘mu’ rhythm) and 16-20 Hz (the mid-beta band). These frequency bands also showed higher phase coherence in the inferior parts of subcentral gyrus and opercular part of the inferior frontal gyrus in the left hemisphere; The 16-20 Hz also extended to the premotor cortex. The specific involvement of sensory and motor systems in the mu and beta bands were consistent with the alpha-beta synchronization in motor control and motor imagery (e.g., McFarland, Miner, Vaughan, & Wolpaw, 2000; Pfurtscheller, Brunner, Schlögl, & Da Silva, 2006), suggesting the specific dynamics for the motor simulation and somatosensory estimation in the motor-to-sensory transformation (Tian & Poeppel, 2010, 2012; Tian et al., 2016). The right hemisphere dominance was consistent with imagery in a singing context (e.g., Özdemir, Norton, & Schlaug, 2006; Riecker, Ackermann, Wildgruber, Dogil, & Grodd, 2000; Zarate, 2013).**

**In the low-gamma band, greater synchronization was observed in the center-group than that in the dispersed-group over the entire motor and sensory systems that showed tracking of imagery in the delta band (Fig. 2D). These results are consistent with the view of local computations for the gamma band (Buzsáki & Wang, 2012; Kopell, Ermentrout, Whittington, & Traub, 2000). The distinctive neural involvement in the tracking rate of the delta, as well as in the theta, mu, beta, and gamma bands, collaboratively indicates the extent of the motor-to-sensory transformation network and the specific functional dynamics of each frequency component in this network.’]**

**In Discussion, [‘The observations of neural structures that operate at separate frequency bands are consistent with our hypothesis regarding the algorithms and neural implementations in the motor-to-sensory transformation. First, using songs with a rhythm in the delta range, a complete frontal-parietal-temporal network was revealed in the corresponding low-frequency band during the imagined singing tasks [Fig. 2D]. These results provide functional evidence suggesting the extent of the motor-to-sensory transformation pathway, consistent with findings in neuroimaging studies (Hurlburt, Alderson-Day, Kühn, & Fernyhough, 2016; McGuire et al., 1995; McGuire et al., 1996). More importantly, the neural phase tracking in the delta band suggested that the fluctuation of the excitability in the network temporally aligned with the unfolding of mental operations.**

**Second, phase coherence at the theta band was localized at temporal auditory regions [Fig. 3A]. These results are consistent with the view that theta oscillations actively mediate the auditory and speech processes (Teng et al., 2018; Teng et al., 2017). In the context of motor-to-sensory transformation, the precise localization of the theta band suggested that theta oscillations could mediate specific computations for constructing speech-like representations.**

**Third, the mu and beta bands have indicated neural rhythmic signatures for both motor control and imagery (e.g., McFarland et al., 2000; Pfurtscheller et al., 2006). In this study, we observed a broad sensorimotor network operating in the mu and beta rhythms during the imagined singing tasks [Fig. 3B&D]. These results further suggested that computations could be executed at these rhythms to internally simulate action during imagery (Li et al., 2020; Ma & Tian, 2019; Tian & Poeppel, 2010, 2012; Tian et al., 2016). Such motor simulations presumably generate an efference copy that is used to emulate the somatosensory consequences of actions for estimating the status of motor effectors (Tian & Poeppel, 2010, 2012; Tian et al., 2016).**

**The hemispheric lateralization highlighted the commonalities and distinctions between inner speech and covert singing. The bilateral observations in the delta band supported neural entrainment to the common rhythmicity in both types of mental operations. The lateralized theta band in the left auditory cortices was consistent with speech-weighted processing (Giraud & Poeppel, 2012). Whereas, the right-hemispheric dominance in the mu and beta bands over the sensorimotor cortices suggested the potential construction of melodic and tonal information in the singing context (e.g., Özdemir et al., 2006; Riecker et al., 2000; Zarate, 2013). The switching from right to left hemisphere is consistent with the observations that the contents of auditory information determine the hemispheric lateralization –the right-lateralized processing of the humming tones is switched to the left lateralization when the tones are superimposed on syllabic contents (Mei, Flinker, Zhu, Cai, & Tian, 2020).**

**Fourth, the gamma band was observed over the entire motor and sensory systems. This result indicated the implementation of local computations in modality-specific regions (Buzsáki & Wang, 2012; Kopell et al., 2000). Together, the motor-to-sensory transformation network possesses distinct dynamics for mediating mental operations. The frontal-parietal-temporal network entrains the rhythm of metal operations. This low-frequency entrainment is suggested to facilitate communication and coordination between cortical areas for specific tasks via traveling waves (Arnal, Doelling, & Poeppel, 2015; Muller, Chavane, Reynolds, & Sejnowski, 2018; Nácher, Ledberg, Deco, & Romo, 2013). Moreover, via cross-frequency coupling (Buzsáki & Wang, 2012), specific frequencies (theta for auditory, mu and beta for sensorimotor) may modulate the gamma band to achieve the computations of motor simulation as well as somatosensory and auditory estimation in the motor-to-sensory transformation in the imagery tasks (Giraud & Poeppel, 2012; Kopell et al., 2000). Future studies are necessary to investigate these hypotheses regarding the connectivity across frequencies and cortical regions so that the functional contributions of the proposed model can be thoroughly examined.’]**

I only have minors comments regarding the methodology and the main text that I list below :

- The term Reaction Time (RT) is confusing, the authors could consider using a more explicit term that state that this metric meaure the duration of imagination period

**The reaction time for imagery was defined as the duration from the onset of imagery go cue (fixation changed color) until participants’ button-press that indicated the finish of the imagery task. We have added clarifications in the Method section.**

- In a related vein, the sentence « We found that when participants

imagined with less temporal variation … » can be reformulated in order to clarify that it concerns the variation of the duration of the imagination period accross trials

**Thank you for this suggestion. We have updated the statement in the Abstract to ‘We found that when participants performed imagery tasks in similar durations across trials, ….’**

- Could the authors justify why they used ICA instead of Signal Space Projection (SSP) for correction of eye movement and cardiac artifacts?

**We agree with the reviewer that both ICA and SSP are two reliable and widely used approaches to remove eye movement artifacts. The effects of artifact rejection should be on par for both methods. Some studies found that ICA had a better balance between artifact rejection and retaining signals than SSP [e.g. Haumann, N. T., Parkkonen, L., Kliuchko, M., Vuust, P., & Brattico, E. (2016). Comparing the performance of popular MEG/EEG artifact correction methods in an evoked-response study. *Computational Intelligence and Neuroscience*, *2016*.]. Considering the novel nature of this study as well as that potentially less power is in imagery compared to perception, we chose to use ICA.**

- « Furthermore, eight trails were randomly sampled from 24 trials of each song and yielded a new group of 24 trials (between-group) » : change trails by trials

**Thank you. We have corrected the typo.**

- “Comparing the phase coherence results in imagined singing with those in listening conditions, the observations were overlapped in the temporal regions of primary and secondary auditory cortices. » The authors did not compare the listening and imagination conditions and did not estimate the conjunction (overlap) between these conditions. These analyses should be added if the authors want to make this statement.

**We thank the reviewer for pointing this out. According to this excellent suggestion, we have conducted two additional analyses -- directly comparing between listening and imagery conditions, as well as a conjunction analysis between conditions. The contrasts show that stronger phase coherence in the HG and pINS in the listening condition than that in the imagery condition, whereas greater phase coherence in the premotor areas and IPS in the imagery condition than that in the listening condition. These results were consistent with common observations that perception has a more robust neural activation than imagery and additional frontal and parietal areas are engaged in speech imagery (e.g., Tian & Poeppel, 2010). Moreover, in the conjunction analysis, we found that the significant phase coherence in listening and imagery conditions overlapped over the primary auditory cortex and extended to the posterior part of the insular cortex and in the anterior part of STG. These new results support our original conclusions. We have added these new results as Fig. 2 in the Results section and copied below.**

**In the Results section, [‘The direct comparison between listening and imagery revealed stronger phase coherence in the HG and pINS in the listening condition than that in the imagery condition [Fig. 2E], whereas greater phase coherence in the premotor areas and IPS in the imagery condition than that in the listening condition [Fig. 2F]. These results were consistent with common observations that perception has a more robust neural activation than imagery and additional frontal and parietal areas are engaged in speech imagery (e.g., Tian & Poeppel, 2010). More importantly, in the conjunction analysis, the significant phase coherence in listening and imagery conditions overlapped over the primary auditory cortex and extended to the posterior part of the insular cortex and in the anterior part of STG [Fig. 2G]. These overlaps suggest the motor-to-sensory transformation during imagery can induce similar auditory representation as in perception (Tian et al., 2018).’ ]**

Reviewer #3: Li and colleagues use a clever design to study where in the brain we can find phase-synchronised activity while participants imagine to sing a few lines of children’s songs. Significant phase coherence in the delta band can be found in a large network comprising bilateral inferior frontal, motor, temporal, and parietal areas. The analysis is new in the context of imagined singing (though standard for speech studies). The study has the potential to inform about the neural representation of imagined singing, but I have a few major (essential) and minor concerns and questions.

**We thank the reviewer for acknowledging the significance and novelty of our study. According to the constructive comments, we have reframed and broadened the research question, provided an explicit rationale for the predetermined sample size, conducted additional analyses, and provided clarifications throughout the manuscript. Especially, the new analyses in more frequency bands provide crucial evidence supporting the suggested reframed research questions regarding inner speech and covert singing. (We really appreciate these suggestions. They elevate our study to a new level. ) Please find our detailed response below.**

Major

1. The study is framed in a “verbal thinking” or “inner speech” context (as indicated by title, abstract, introduction and discussion), but I don’t think it is appropriate to equate thinking with singing. Singing (production and perception) involves a wider/different network than thinking. The right hemisphere for example, is much more involved in singing than verbal thinking. In my opinion, the manuscript needs to reflect these basic differences and the topic needs to be reframed.

**We really appreciate the excellent suggestion. Our original focus was on the inner speech of verbal thinking, and the implementation of signing was more on the methodological side to reduce the temporal variance of behavior and increase the statistical power of ITC. After reading this comment, we realized that the dynamics of covert singing could be also another important aspect. According to the excellent suggestion, we have updated our manuscript throughout. We now use ‘mental operations’ to unify the inner speech and covert singing as two mental processes of vocal production that possess similar dynamics. This highlights the similarity of rhythmicity in both inner speech and covert singing in the findings of neural entrainment to the inner rhythm.**

**We fully agree with the reviewer that singing involves a wider/different neural network, especially in the right hemisphere. Our study could potentially reveal the differences between inner speech and covert singing. Consistent with this goal, our new analysis and results (the new Fig. 3) revealed different hemispheric lateralization in distinct frequencies. The theta band was observed in the left hemisphere, consistent with the view of more speech processing [Giraud, A. L., & Poeppel, D. (2012). Cortical oscillations and speech processing: emerging computational principles and operations. *Nature neuroscience*, *15*(4), 511.]. Whereas the mu and beta bands were more in the right hemisphere, suggesting the potential construction of melodic and tonal information in the singing context [e.g. Riecker, A., Ackermann, H., Wildgruber, D., Dogil, G., & Grodd, W. (2000). Opposite hemispheric lateralization effects during speaking and singing at motor cortex, insula and cerebellum. *Neuroreport*, *11*(9), 1997-2000.; Özdemir, E., Norton, A., & Schlaug, G. (2006). Shared and distinct neural correlates of singing and speaking. Neuroimage, 33(2), 628-635.; Zarate, J. M. (2013). The neural control of singing. *Frontiers in human neuroscience*, *7*, 237.]. We have included the new results as Fig. 3, and updated the Results section and copied below.**

**In Results, [‘To further explore the functional specificity of dynamic processing in the motor-to-sensory pathway, we performed the phase-coherence analysis in a broad range of frequency bands [Fig. 3]. We found that in the 4-7 Hz (the theta band), only the primary auditory cortex and anterior part of STG and STS in the left hemisphere showed significant phase coherence. This focused theta band activation in the auditory cortices, contrasted with the collaborative frontal-parietal-temporal network activation of the entire motor-to-sensory pathway in the delta band (Fig. 1), was consistent with the specific role of the theta band in auditory and speech processing (e.g., Teng, Tian, Doelling, & Poeppel, 2018; Teng et al., 2017).**

**A broad sensorimotor network in the right hemisphere was observed in 8-12 Hz (the alpha band, or the ‘mu’ rhythm) and 16-20 Hz (the mid-beta band). These frequency bands also showed higher phase coherence in the inferior parts of subcentral gyrus and opercular part of the inferior frontal gyrus in the left hemisphere; The 16-20 Hz also extended to the premotor cortex. The specific involvement of sensory and motor systems in the mu and beta bands were consistent with the alpha-beta synchronization in motor control and motor imagery (e.g., McFarland, Miner, Vaughan, & Wolpaw, 2000; Pfurtscheller, Brunner, Schlögl, & Da Silva, 2006), suggesting the specific dynamics for the motor simulation and somatosensory estimation in the motor-to-sensory transformation (Tian & Poeppel, 2010, 2012; Tian et al., 2016). The right hemisphere dominance was consistent with imagery in a singing context (e.g., Özdemir, Norton, & Schlaug, 2006; Riecker, Ackermann, Wildgruber, Dogil, & Grodd, 2000; Zarate, 2013).**

**In the low-gamma band, greater synchronization was observed in the center-group than that in the dispersed-group over the entire motor and sensory systems that showed tracking of imagery in the delta band (Fig. 2D). These results are consistent with the view of local computations for the gamma band (Buzsáki & Wang, 2012; Kopell, Ermentrout, Whittington, & Traub, 2000). The distinctive neural involvement in the tracking rate of the delta, as well as in the theta, mu, beta, and gamma bands, collaboratively indicates the extent of the motor-to-sensory transformation network and the specific functional dynamics of each frequency component in this network.’]**

2. Related to 1: I imagine that the same pattern of results would arise if participants were to just imagine humming the song, keeping the rhythmicity. I don’t think the results necessarily show processes related to “inner thinking” or speech, but they show consistent neural processes related to the rhythmicity and tonality of imagining a song. This should at least be discussed.

**Again, we fully agree with the reviewer. Continuous with our responses to Comment #1, we have added new paragraphs about our new results in the Discussion section to highlight the common aspect of rhythmicity, as well as the distinctions of speech-weighed and tonality-weighed in these two mental operations of vocal production. We copied the major updates below.**

**In Discussion, [‘The observations of neural structures that operate at separate frequency bands are consistent with our hypothesis regarding the algorithms and neural implementations in the motor-to-sensory transformation. First, using songs with a rhythm in the delta range, a complete frontal-parietal-temporal network was revealed in the corresponding low-frequency band during the imagined singing tasks [Fig. 2D]. These results provide functional evidence suggesting the extent of the motor-to-sensory transformation pathway, consistent with findings in neuroimaging studies (Hurlburt, Alderson-Day, Kühn, & Fernyhough, 2016; McGuire et al., 1995; McGuire et al., 1996). More importantly, the neural phase tracking in the delta band suggested that the fluctuation of the excitability in the network temporally aligned with the unfolding of mental operations.**

**Second, phase coherence at the theta band was localized at temporal auditory regions [Fig. 3A]. These results are consistent with the view that theta oscillations actively mediate the auditory and speech processes (Teng et al., 2018; Teng et al., 2017). In the context of motor-to-sensory transformation, the precise localization of the theta band suggested that theta oscillations could mediate specific computations for constructing speech-like representations.**

**Third, the mu and beta bands have indicated neural rhythmic signatures for both motor control and imagery (e.g., McFarland et al., 2000; Pfurtscheller et al., 2006). In this study, we observed a broad sensorimotor network operating in the mu and beta rhythms during the imagined singing tasks [Fig. 3B&D]. These results further suggested that computations could be executed at these rhythms to internally simulate action during imagery (Li et al., 2020; Ma & Tian, 2019; Tian & Poeppel, 2010, 2012; Tian et al., 2016). Such motor simulations presumably generate an efference copy that is used to emulate the somatosensory consequences of actions for estimating the status of motor effectors (Tian & Poeppel, 2010, 2012; Tian et al., 2016).**

**The hemispheric lateralization highlighted the commonalities and distinctions between inner speech and covert singing. The bilateral observations in the delta band supported neural entrainment to the common rhythmicity in both types of mental operations. The lateralized theta band in the left auditory cortices was consistent with speech-weighted processing (Giraud & Poeppel, 2012). Whereas, the right-hemispheric dominance in the mu and beta bands over the sensorimotor cortices suggested the potential construction of melodic and tonal information in the singing context (e.g., Özdemir et al., 2006; Riecker et al., 2000; Zarate, 2013). The switching from right to left hemisphere is consistent with the observations that the contents of auditory information determine the hemispheric lateralization –the right-lateralized processing of the humming tones is switched to the left lateralization when the tones are superimposed on syllabic contents (Mei, Flinker, Zhu, Cai, & Tian, 2020).’]**

3. The phase synchronisation results during Listening and Imagery are compared in the discussion, but a statistical comparison in the results is missing. This is the most important contrast in this study (to find out what the “inner” aspect of singing actually adds/changes when compared to simply listening to songs). This could for example be done on a trial-by-trial basis.

**We thank the reviewer for pointing this out. According to this excellent suggestion, we have conducted two additional analyses -- directly comparing between listening and imagery conditions, as well as a conjunction analysis between conditions. The contrasts show that stronger phase coherence in the HG and pINS in the listening condition than that in the imagery condition, whereas greater phase coherence in the premotor areas and IPS in the imagery condition than that in the listening condition. These results were consistent with common observations that perception has a more robust neural activation than imagery and additional frontal and parietal areas are engaged in speech imagery (e.g., Tian & Poeppel, 2010). Moreover, in the conjunction analysis, we found that the significant phase coherence in listening and imagery conditions overlapped over the primary auditory cortex and extended to the posterior part of the insular cortex and in the anterior part of STG. These new results support our original conclusions. We have added these new results as Fig. 2 in the Results section and copied below.**

**[‘The direct comparison between listening and imagery revealed stronger phase coherence in the HG and pINS in the listening condition than that in the imagery condition [Fig. 2E], whereas greater phase coherence in the premotor areas and IPS in the imagery condition than that in the listening condition [Fig. 2F]. These results were consistent with common observations that perception has a more robust neural activation than imagery and additional frontal and parietal areas are engaged in speech imagery (e.g., Tian & Poeppel, 2010). More importantly, in the conjunction analysis, the significant phase coherence in listening and imagery conditions overlapped over the primary auditory cortex and extended to the posterior part of the insular cortex and in the anterior part of STG [Fig. 2G]. These overlaps suggest the motor-to-sensory transformation during imagery can induce similar auditory representation as in perception (Tian et al., 2018).’]**

4. Related to the previous point – there were two different alpha levels used for analysis in the Listening and Imagery conditions, so their results can’t be compared. I.e. just before the result section: “(alpha=0.05 for the listening conditions, and alpha=0.001 for the imagery conditions).” In the result section itself, there’s even another alpha level mentioned: “For the imagery condition, [..] (pcorr(FDR)<0.01)”. The same thresholds should be used for all conditions, unless there is a clear justification for this.

**In the previous version, the p-values differed between the listening and imagery conditions because different statistical procedures were used. In the listening condition, first, a random sample grouping of between-groups was conducted 100 times to increase the reliability of generated baselines. The 95th percentile of ITC values was selected as a baseline value (already a threshold of 0.05) to contrast with the empirical value in the within-groups. Therefore, the p-value 0.05 was selected in the following cluster analysis for the listening condition. Whereas, in the imagery condition, the center-group was directly compared with the dispersed-group in the cluster analysis, so that p-value of 0.001 was used. These above-mentioned p-values are all pre-cluster level threshold. (We did not make it clear in the previous version, and we have clarified in the updated version.) The cluster-level threshold for both conditions are FDR corrected p < 0.05. (The p_corr(FDR) < 0.01 in the main text of the previous version was a typo and we have corrected it.)**

**We agree with the reviewer that the different p-values were confusing. Therefore, in the updated version, we used a similar statistical procedure and the same pre-cluster p-values of 0.001 and cluster level p-values of 0.05 in the cluster analysis for both the listening and imagery conditions. Specifically, we selected the median ITC value (50th percentile) in the 100 random sample grouping of between groups as a baseline value in the listening condition, instead of 95th percentile. The same original procedure was used in the imagery condition. Therefore, the same pre-cluster p-values of 0.001 and cluster level p-values of 0.05 were used in both the listening and imagery conditions. The new results were consistent with the original results. We have clarified these points in the Method section and copied below.**

**[‘The empirical statistics were first obtained by a two-tailed paired t-test with two or more adjacent significant vertices with a pre-cluster threshold of alpha = 0.001. Next, a null distribution was formed by randomly shuffling the group labels 1000 times. Cluster-level, FDR corrected results were obtained by comparing the empirical statistics with the null distribution (cluster threshold of alpha=0.05 for both listening and hearing conditions).’]**

5. The study had only 16 participants (and no a priori estimated sample size), which is becoming less and less acceptable in psychological and neuroimaging studies (e.g. Simmons JP, Nelson LD, Simonsohn U (2011) False-positive psychology: Undisclosed flexibility in data collection and analysis allows presenting anything as significant. Psychological science 22: 1359-1366. <https://doi.org/10.1177/0956797611417632>). While it would be unfair to request additional data, a sample size of 16 is not something that should be advocated, particularly in a wide-reaching journal like Plos Biology. Please add an explicit justification for this low number of participants to demonstrate awareness that this is not good scientific practice.

**We fully agree with the reviewer about the importance of sample size. In fact, we did predetermine our sample size to be 16, based on our previous MEG studies that investigated the similar questions of speech imagery -- both Tian et al., 2018 *Nature Human Behaviour* and Tian & Poeppel, 2015 *J. Cognitive Neuroscience* papers had the same sample size of 16. (Sorry that we did not state it explicitly in the previous version.) Moreover, compared to behavioral measures, MEG is a multi-dimensional tool that is especially sensitive to neural dynamics (also very costly). The neural entrainment to speech is well documented in previous MEG studies. The reliability of this neural measure makes studies with fewer data acceptable in prestigious journals, such as 8 participants in each experiment in Ding et al., 2016 *Nature Neuroscience*, 13 participants in Teng et al., 2020 *Current Biology*, 10 participants in Luo, Liu, & Poeppel, 2010 *PloS Biology*, and 16 participants in Teng, Tian, Rowland, & Poeppel, 2017 *PloS Biology*. (The sample size for previous neural entrainment MEG experiments was typically between 3 and 12 as reported in Ding et al., 2016 *Nature Neuroscience*.) Therefore, our sample size is also consistent with previous studies.**

**We further calculate the effect size that is needed to have 80% power at an alpha level of 0.05. The required effect size for a sample size of 16 is Cohen’s d = 0.99. The post-hoc effect size from our data in all sensors is d = 1.50 (dz = 2.32). In the clustering analysis using in this study, we used a pre-cluster threshold of 0.001 in the paired t-test. Consider the sample size of 16, the minimal effect size is dz = 4.073/sqrt(16) = 1.02. The post-hoc effect size from the data in Heschl’s gyrus that is independently defined by cortical parcellation (S1 Table and S1 Fig.), is d = 1.21 (dz = 1.89). The post-hoc effect sizes obtained in multiple analyses are greater than the required effect size. Therefore, using the predetermined sample size of 16 that is consistent with previous studies should provide enough statistical power to reliably measure the well-documented neural entrainment effects using MEG.**

**Following the reviewer’s excellent suggestion and the guidelines in the suggested paper, we have added an explicit statement in the Method section that we predetermined the sample size based on previous research, and evidence that our study should have enough power, which are copied below.**

**[‘The number of participants was pre-determined based on our previous studies that investigated similar speech imagery using MEG (Tian et al., 2018; Tian & Poeppel, 2015). Moreover, the number of 16 participants is in the upper range of the total participants in previous MEG studies that investigated neural entrainment (e.g., Ding et al., 2016; Luo, Liu, & Poeppel, 2010; Teng et al., 2020; Teng, Tian, Rowland, & Poeppel, 2017). The effect size that is required to have 80% power at an alpha level of 0.05 for a sample size of 16 is Cohen’s d = 0.99. As the standard deviation estimated in our study was 0.07, the absolute effect size in the measure (phase coherence) was 0.07.** **The post-hoc effect size from our data in all sensors was d = 1.50 (dz = 2.32). The absolute effect size is 0.09.** **In the clustering analysis using in this study, we used a pre-cluster threshold of 0.001 in the paired t-test. Consider the sample size of 16, the minimal effect size is dz = 1.02. The post-hoc effect size from the data in Heschl’s gyrus that was independently defined by cortical parcellation (S1 Table and S1 Fig.), was d = 1.21 (dz = 1.89). The absolute effect size is 0.11.** **The post-hoc effect sizes obtained in multiple analyses are greater than the required effect size. Therefore, using the predetermined sample size of 16 that is consistent with previous studies should provide enough statistical power to reliably measure the well-documented neural entrainment effects using MEG.’]**

6. The authors propose a model (Figure 1f) that describes Articulatory Simulation, Articulatory Estimation, and Auditory Estimation, including three different areas involved in these processes. While the results support that these areas (and others) show increased delta phase coherence, none of the three proposed processes can be disentangled, using the present analysis. It might be possible to gain support for some aspects of the model using additional analyses. For example, using directed connectivity, the proposed direction of information flow could be supported. But the present paradigm cannot provide support for the different processes of the model. Please make it clear in the manuscript that the used approach is not suitable to study the different functional contributions of the proposed model (only the involvement of the areas in delta phase alignment).

**We fully agree with the reviewer. We proposed this model in 2010. In ten years, we have conducted a series of studies and progressively investigated the details of each component in this model. This study is our first attempt to collectively reveal the dynamics in the entire proposed network. Although the original results showed the delta band neural entrainment and the new analysis revealed a double dissociation between the operating frequencies and proposed regions, we still need stronger evidence, such as directed connectivity, to further support the proposed functional contributions and information flow in this network. The reviewer correctly points out that the current paradigm is not optimal for this purpose. We have explicitly elucidated this point in the Discussion section and copied below.**

**[‘Together, the motor-to-sensory transformation network possesses distinct dynamics for mediating mental operations. The frontal-parietal-temporal network entrains the rhythm of metal operations. This low-frequency entrainment is suggested to facilitate communication and coordination between cortical areas for specific tasks via traveling waves (Arnal, Doelling, & Poeppel, 2015; Muller, Chavane, Reynolds, & Sejnowski, 2018; Nácher, Ledberg, Deco, & Romo, 2013). Moreover, via cross-frequency coupling (Buzsáki & Wang, 2012), specific frequencies (theta for auditory, mu and beta for sensorimotor) may modulate the gamma band to achieve the computations of motor simulation as well as somatosensory and auditory estimation in the motor-to-sensory transformation in the imagery tasks (Giraud & Poeppel, 2012; Kopell et al., 2000). Future studies are necessary to investigate these hypotheses regarding the connectivity across frequencies and cortical regions so that the functional contributions of the proposed model can be thoroughly examined.’]**

Minor

1. The language in the manuscript is problematic. I understand that it can be extremely difficult as a non-native speaker to write in English and reviewers should as much as possible try to ignore this. But in the current manuscript it is often unclear whether a statement refers to previous research, the current study, or something that is proposed here (due to the unclear use of tenses). Please ask a native speaker to proofread the ms before re-submission.

**We really appreciate the reviewer for sympathizing with our difficulties in writing as non-native speakers. We completely agree with the reviewer that we need to improve the clarity of our manuscript. Therefore, we have asked two native English speakers to proofread our manuscript. If the reviewer still thinks that we need to further improve our writing, we will hire a professional proofreading service to correct our writing so that our novel study and interesting findings can be better delivered to readers.**

2. Please add the fourth song to the analysis, even if is also used for a different research question. With only 16 participants, this study needs as much data as possible.

**The reason for excluding the ‘Twinkle Twinkle Little Star’ from our original analysis was because it had an identical melody with the ‘Alphabet song’. We intended to use these two songs that had the same melody but different lyrics to probe the different processes of melody and tonality (presumably over the right hemisphere) and speech (presumably over the left hemisphere) in both listening and imagery. Nevertheless, we followed the reviewer’s suggestion and included all four songs in the analysis. The results are mostly consistent with our original results with three songs. In the listening condition, the phase coherence was concentrated over the left auditory cortices, extending to the insular cortex. However, the right hemisphere did not show any significant phase coherence. This activation over left hemisphere and no significance over the right counterparts are consistent with our hypothesis -- the same melody of two songs increase the phase coherence in the ‘between-group’ (the null hypothesis generated by combining different songs), and hence the phase coherence difference between ‘within-group’ and ‘between-group’ is no longer significant in the right hemisphere. The phase coherence localization results of imagery using four songs are similar to those using three songs, but with less power presumably because of the same reason of similar melody between two songs. We copied the results below. We did not include these new results in the manuscript, because it was not the goal of this paper and could distract readers. But if the reviewer deems it is necessary, we will add them to the supplementary information.**

3. In the introduction, particularly on page 3, a lot of information is missing at this point to understand the statements here. It is unclear what is meant with “reaction time” or what the conditions are. The descriptions here don’t make sense without knowing the paradigm. Please explain briefly what the participants had to do (maybe at the beginning of the last paragraph of the intro).

**Thank you for this suggestion. We have added a brief description of our experiment at the beginning of the last paragraph in the Introduction. We also changed ‘reaction time’ to ‘durations of performing imagery tasks’ so that it can directly link to our experiment.**

4. Please add page and line numbers to the manuscript.

**We have added page and line numbers in our manuscript.**

5. The approach used here is described as “frequency tagging”. Frequency tagging is well defined in the field and I think neither the listening nor (even less) the imagery condition can be classed as frequency tagging.

**We agree with the reviewer that the classic term of ‘frequency tagging’ may not accurately describe our novel paradigm. Therefore, according to the suggestion in a recent review [Obleser, J., & Kayser, C. (2019). Neural entrainment and attentional selection in the listening brain. Trends in cognitive sciences, 23(11), 913-926.], we renamed our approach as ‘rhythmic entrainment paradigm’, with ‘entrainment in the broad sense’.**

6. In the figure, please indicate the areas in the result plots (1i and 1j) that refer to proposed areas in 1f, to make a comparison of hypotheses and results easier.

**This is a good idea. In the updated version, we have linked the results plots (the original Fig. 1i&j, now the new Fig. 2C&D) with the model by referring to the proposed areas in Fig. 1F.**

7. Please use a more informative plot for 1b. Bar plots are becoming unacceptable in scientific research.

**We have added individual reaction time data in Fig. 1b and copied below.**

8. Source data were spatially smoothed – using which parameters?

**We have added this information in the Method section.**

**[‘Source data were spatially smoothed using a Gaussian smoothing function 'SurStatSmooth' in the SurStat toolbox [Worsley et al., 2009] with the parameter of FWHM (Full width at half maximum) of 3mm.’]**

9. Please define the terms of the equation (i.e. t,f,N,θ). Should the θ be δ?

**The greek letter** θ **is a common notion representing angle. Here we use it to represent the phase angle. To avoid confusion with the frequency bands, we now use all spell-out words (e.g. delta, theta) for the frequency bands. Moreover, we have provided definitions for all parameters in Eq. 1.**

**[‘In Eq. 1, *θ_j_ (t,f)* represents the phase value at time point *t* and frequency *f* in the *j*th trial. *N* represents the total number of trials in a group.’]**

10. Why were only 4 s post-stimulus analysed? The songs were a minimum of 5.42 s long, and participants needed consistently longer to imagine the songs. I would think that 6 s post-stimulus would maximise available data for each epoch, and I would also add an additional 2 s (consistent with the pre-stimulus time) to optimise the FFT results (leading to 10 s [-2s to 8 s] epochs).

**The duration of imagery across trials and among participants could vary over a wide range. The imagery durations in some trials, especially for the shortest song, could be shorter than the length of epochs if the epochs were extracted too long. To be conservative in this novel study, we had chosen a stringent threshold of 4s. We agree with the reviewer that it is probably fine to include 6s data -- the more data points may balance the reduction of statistical power due to the variance of imagery durations across trials. Therefore, we followed the constructive suggestion and used 10s data in FFT (2s of pre-stimulus and 8s post-stimulus) and applied the same analysis with the 6s post-stimulus data. The new results are consistent with our original results. We have added these results in the Supplementary information S2 Fig. and copied below.**
