# Supplementary material for: Mental operations in rhythm: Motor-to-sensory transformation mediates imagined singing
Source: PLoS Biol. 2020 Oct 5;18(10):e3000504. doi: 10.1371/journal.pbio.3000504 (PMC7561264; doi:10.1371/journal.pbio.3000504)
Supplement: Attachment — Submitted filename: response to reviewers.docx [file pbio.3000504.s006.docx]

Dear Dr. Gasque,

We thank you and the reviewers again for your constructive comments. We are glad to hear that we have addressed all the major comments. In this round of revisions, we have made changes accordingly. Specifically, we have added a brief discussion about the ITC differences between imagery and hearing in multiple frequency bands, since numerous studies of speech tracking have provided evidence that differs from imagery tracking observed in this study. Moreover, we have updated and expressed a statement more carefully to explicitly highlight its speculative nature. Furthermore, we have deleted the circular argument that was identified by the statistical analysis expert. We have also made several technical changes according to the guidance of formatting requests.

Sincerely,

Xing Tian

(on behalf of Yanzhu Li and Huan Luo)

------------------------------------------------------------------------
Reviewer remarks:

Reviewer #1, Thomas Whitford: The authors have considered my comments carefully and made detailed responses. I have no further comments.

RE: We are glad to hear that we have addressed all the reviewer’s comments.

Reviewer #2: I thank the authors for this revised manuscript and for taking my comments into account. The article has been greatly improved.
 I have one final comment concerning the last analysis: The ITC for the theta, mu, beta and gamma bands has been computed only for the imaginary condition: in order to be able to conclude that these different oscillatory dynamics (and networks) are specific to mental imagery a direct contrast between the imaginary and listening condition (for each frequency band) is needed.

RE: we appreciate the reviewer’s comment. The focus of this study is to investigate the motor-to-sensory transformation during mental operations. Moreover, numerous studies have demonstrated that the ITC during listening is absent in mu, and beta bands, which are different from our observations during imagery. We think that it would be unnecessary to repeat the results that have been replicated many times. We have added a discussion about the current finding in imagery compared with previous findings in perception in the Discussion section and copied below.

‘The phase coherence and its distribution during imagery are distinct from previous observations in speech perception (e.g. der Nederlanden, Joanisse, & Grahn, 2020; Luo & Poeppel, 2007, 2012; Teng & Poeppel, 2020; Teng et al., 2018; Teng et al., 2017; Wang et al., 2012), suggesting unique motor-to-sensory transformation during mental operations.’

References:

der Nederlanden, C. M. V. B., Joanisse, M., & Grahn, J. A. (2020). Music as a scaffold for listening to speech: Better neural phase-locking to song than speech. *NeuroImage*, 116767.

Luo, H., & Poeppel, D. (2007). Phase patterns of neuronal responses reliably discriminate speech in human auditory cortex. *Neuron, 54*(6), 1001-1010.

Luo, H., & Poeppel, D. (2012). Cortical oscillations in auditory perception and speech: evidence for two temporal windows in human auditory cortex. *Frontiers in Psychology, 3*, 170.

Teng, X., & Poeppel, D. (2020). Theta and Gamma bands encode acoustic dynamics over wide-ranging timescales. *Cerebral Cortex, 30*(4), 2600-2614.

Teng, X., Tian, X., Doelling, K., & Poeppel, D. (2018). Theta band oscillations reflect more than entrainment: behavioral and neural evidence demonstrates an active chunking process. *European Journal of Neuroscience, 48*(8), 2770-2782.

Teng, X., Tian, X., Rowland, J., & Poeppel, D. (2017). Concurrent temporal channels for auditory processing: Oscillatory neural entrainment reveals segregation of function at different scales. *PLoS biology, 15*(11).

Wang, Y., Ding, N., Ahmar, N., Xiang, J., Poeppel, D., & Simon, J. Z. (2012). Sensitivity to temporal modulation rate and spectral bandwidth in the human auditory system: MEG evidence. *Journal of neurophysiology, 107*(8), 2033-2041.

Reviewer #3: I would like to thank the authors for their thorough reply and the additional analyses, in particular the statistical contrast between conditions and the conjunction plot. I also like the inclusion of the other frequency bands. Overall, my comments have been adequately addressed.

The only thing I would like to add after reading the revision is that I would be very careful with the idea that the current study can highlight "commonalities and distinctions between inner speech and covert singing" (highlighted manuscript: page 25/line 3, clean manuscript: page 24/line 22). It is true that nursery rhymes are somewhere in between these two stimulus types, but I don't think that it can highlight differences and commonalities between inner speech and covert singing. To do this, it would be necessary to use these two types of stimuli/tasks separately. Any conclusions regarding the differences/similarities based on this used stimulus type must remain speculative (which is okay in a discussion). Also, these conclusions use "reverse-inferring" based on previously found brain regions and this is usually selective and not necessarily informative or applicable. Could the authors express their thoughts on page 25 more carefully and explicitly highlight that this is
speculative?

RE: We thank and agree with the reviewer’s comment. We have changed this statement into ‘The hemispheric lateralization suggests the possible commonalities and distinctions between inner speech and covert singing’.
